# LC-ESI-MS/MS Characterization of Concentrated Polyphenolic Fractions from *Rhododendron luteum* and Their Anti-Inflammatory and Antioxidant Activities

**DOI:** 10.3390/molecules27030827

**Published:** 2022-01-26

**Authors:** Lena Łyko, Marta Olech, Renata Nowak

**Affiliations:** Department of Pharmaceutical Botany, Medical University of Lublin, 1 Chodźki Street, 20-093 Lublin, Poland; lenalyko18@gmail.com (L.Ł.); renata.nowak@umlub.pl (R.N.)

**Keywords:** polyphenolics, phenolic acids, flavonoids, LC-MS, yellow azalea, honeysuckle azalea, Ericaceae, bioactive plant secondary metabolites

## Abstract

The high biological potential of polyphenols encourages the search for new natural sources of and biomedical applications for these compounds. *Rhododendron luteum* Sweet was previously reported to contain pharmaceutically active polyphenols. The present research investigates the polyphenolic fractions in *R. luteum* leaves, including a determination of the free and bound phenolic acid and flavonoid contents and their anti-inflammatory and antioxidant activities. LC-ESI-MS/MS (liquid chromatography/electrospray ionization triple quadrupole mass spectrometry) analysis revealed a great abundance of free (e.g., 5-*O*-caffeoylquinic acid, ferulic acid, protocatechuic acid, catechin, and dihydromyricetin) and bound (e.g., caffeic acid, *p*-coumaric, protocatechuic acid, myricetin, quercetin) phenolics. The *R. luteum* samples exhibited high anti-inflammatory potential in lipoxygenase (IC_50_: 0.33 ± 0.01–2.96 ± 0.06 mg dry extract (DE)/mL) and hyaluronidase (IC_50_: 78.76 ± 2.09 – 429.07 ± 31.08 µg DE/mL) inhibition capacity assays. Some samples also had the ability to inhibit cyclooxygenase 1 (IC_50_: 311.8 ± 10.95 µg DE/mL) and cyclooxygenase 2 (IC_50_: 53.40 ± 5.07; 608.09 ± 14.78 µg DE/mL). All fractions showed excellent antioxidant activity in the Oxygen Radical Absorbance Capacity (ORAC) assay (5.76–221.81 g Trolox/g DE), ABTS^•+^ radical scavenging ability (0.62 ± 0.03 – 5.09 ± 0.23 g Trolox/g DE), and moderate ion (Fe^2+^) chelating power. This paper expands our knowledge of the phytochemistry and pharmacological activity of *R. luteum* polyphenols. It reveals, for the first time, the presence of dihydromyricetin, afzelin, and laricitrin in the plant material. It indicates biologically active polyphenolic fractions that should be further investigated or which could be efficiently used in pharmaceutical, cosmetic, or nutraceutical applications.

## 1. Introduction

The *Rhododendron* genus (Ericaceae family) comprises over 850 species distributed throughout the world, mainly in the northern hemisphere [1]. Rhododendrons produce secondary metabolites which exert various kinds of activity; many of them have been found to have low toxicity and to be effective in the treatment of chronic inflammation [2]. The main phytochemicals occurring in rhododendron species are polyphenols, especially flavonoids and terpenes [1].

Polyphenols are able to alleviate inflammation by decreasing the levels of interleukins, prostaglandin E2, and TNF-α, as well as modulating enzymes such as lipoxygenase (LOX), cyclooxygenase 2 (COX 2), and cytokine-inducible nitric oxide synthase (iNOS) [3]. Moreover, it has been reported that phenolic-rich extracts are able to inhibit hyaluronidase, i.e., another enzyme that is directly related to inflammation [4,5]. In humans, hyaluronidase is responsible for the degradation of hyaluronic acid into tetrasaccharides, which are associated with pro-inflammatory cytokine release [6].

Widely used nonsteroidal anti-inflammatory drugs cause multiple adverse effects. The long-term administration of these drugs may result in ulceration and damage to gastric mucosa, hepatotoxicity, and hypertension [7]. Hence, the use of polyphenols seems to be a promising alternative.

One of the *Rhododendron* genus representatives is *Rhododendron luteum* Sweet, known as yellow azalea or honeysuckle azalea (lat. *Azalea pontica* L. or *Rhododendron flavum* G. Don; RL). Investigations of Turkish specimens showed many interesting activities, such as inhibition of bacterial growth and cytotoxicity. In addition, extracts of RL leaves exhibited the ability to inhibit enzymes such as acetylcholinesterase, butyrylcholinesterase, tyrosinase, and α-glucosidase [8].

Our previous investigation revealed high total polyphenol and flavonoid contents in different RL extracts. *R. luteum* was also reported to contain many polyphenols which are known for their pharmacological activity [8,9]. However, to date, there is no report on the distribution of free and bound phenolic acids or flavonoid aglycones in *R. luteum* leaves. It has been observed that the total phenolic content in RL extracts correlates with the capacity of scavenging free radicals, although there is no information regarding which polyphenols may be involved in this activity. Similarly, there is only a negligible amount of data about the anti-inflammatory activity of RL extracts and its possible relation to their phenolic profile.

Therefore, we analyzed different *R. luteum* polyphenolic fractions with the objective of determining the free and bound phenolic acids and flavonoids profile. The detailed phenolic composition of purified and concentrated samples was determined with the use of liquid chromatography/electrospray ionization triple quadrupole mass spectrometry (LC-ESI-MS/MS). In the present report, we wanted to investigate the antioxidant and anti-inflammatory potential of *R. luteum*. For this purpose, we examined the LOX, COX-1, COX-2, and hyaluronidase inhibitory ability of all RL samples, their radical scavenging and metal chelating power, and oxygen radical absorbance capacity. We also wanted to indicate RL metabolite/-s with the greatest impact on biological activity.

## 2. Results and Discussion

### 2.1. Chemical Profile of Polyphenolic Fractions

**Figure 1:**
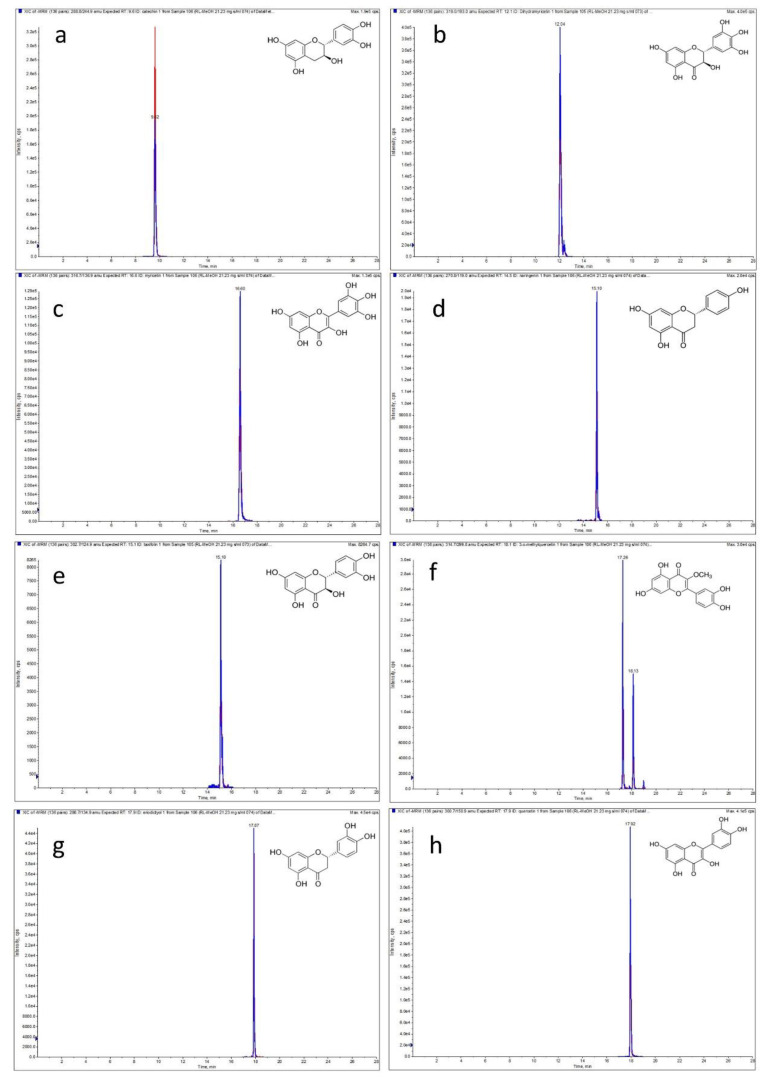
LC-MS chromatograms obtained in the multiple reaction monitoring (MRM) mode of flavonoid aglycones detected in *R. luteum* leaves (sample RL-M). Monitored MRM transitions are given in the brackets: (**a**): catechin (m/z 288.8 → 109; 288.8 → 244.9); (**b**): dihydromyricetin (m/z 319 → 193; 319 → 125); (**c**): myricetin (m/z 316.7 → 136.9; 316.7 → 150.9); (**d**): naringenin (m/z 270.8 → 119; 270.8 → 150.9); (**e**): taxifolin (m/z 302.7 → 124.9; 302.7 → 284.8); (**f**): 3-*O*-methylquercetin (m/z 314.7 → 299.8; 314.7 → 270.8); (**g**): eriodictyol (m/z 286.7 → 134.9, 286.7 → 150.9); (**h**): quercetin (m/z 300.7 → 150.9; 300.7 → 178.8).

Plant metabolites belonging to diverse chemical groups can exhibit different biological and pharmacological activities, e.g., antibacterial, antirheumatoid, antioxidant, anti-inflammatory, antinociceptive, enzyme inhibitory and cytotoxic potential [2,6,10,11]. Our previous research of *Rhododendron luteum* Sweet leaf extracts revealed the presence of active polyphenolic compounds. It was observed that the biological activity of the samples was strongly associated with the content of phenolic acids and flavonoids [9]. Therefore, in this study, we decided to analyze the phenolic acid and flavonoid aglycone profiles of the plant material. For this purpose, concentrated fractions of free and bound phenolic acids and flavonoid aglycones were obtained according to previously optimized phytochemical procedures [12,13]. Moreover, the antioxidant and anti-inflammatory potential of the basic methanolic extract and all polyphenolic fractions was determined. A phytochemical analysis was performed using the LC-ESI-MS/MS-MRM method. This facilitated the detection and quantification of a wide range of analytes belonging to several polyphenol subclasses (Table 1). As a result, the content of five hydroxybenzoic acids (protocatechuic, gallic, gentisic, vanillic, and 4-hydroxybenzoic), four hydroxycinnamic acids (3-*O*-caffeoylquinic, caffeic, *p*-coumaric, and ferulic), twelve flavonoid aglycones (laricitrin, dihydromyricetin, myricetin, catechin, naringenin, taxifolin, luteolin, eriodictyol, quercetin, 3-*O*-metylquercetin, apigenin, and kaempferol) and seven flavonoid glycosides (afzelin, hyperoside, isoquercetin, vitexin, isovitexin, quercitrin, and naringenin 7-*O*-glucoside) was revealed (Figure 1 and Figures S1 and S2 in the Supplementary material).

As shown in Table 1, 5-*O*-caffeoylquinic acid was found to be the main phenolic acid present in the RL-M sample (basic methanolic extract). Its amount was 18.546 ± 0.093 mg/g of dry extract (DE). The presence of other chlorogenic acids, i.e., 3-*O*-caffeoylquinic acid and 4-*O*-caffeoylquinic acid, was also verified; however, these compounds were not detected in the samples. As reported previously, chlorogenic acids, including 5-*O*-caffeoylquinic acid, commonly occur in *Rhododendron* species [14,15,16,17,18,19]. Among phenolic acids, protocatechuic, gallic, *p*-coumaric, and gentisic acids were also present in RL-M, although their concentrations were lower (respectively: 0.397 ± 0.002; 0.267 ± 0.001; 0.087 ± 0.003; 0.016 ± 0.001 mg/g DE). Similar to our previous findings, 4-hydroxybenzoic, caffeic, and ferulic acids were detected in the methanol extract in trace amounts. Vanillic acid was not detected in RL-M.

Catechin (6.081 ± 0.043 mg/g DE) and dihydromyricetin (4.032 ± 0.086 mg/g DE) were the dominant flavonoids in the methanol extract. The catechin content was previously determined in different RL extracts, although the extraction type used in this study yielded higher quantities [9]. Catechin was also identified in other *Rhododendron* representatives, i.e., *R. mucronulatum*, *R. decorum*, *R. ambiguum*, and *R. cinnabainum* [1,14]. It is worth emphasizing that dihydromyricetin (also known as ampelopsin or ampeloptin) was detected in *R. luteum* for the first time in our study. However, there are data about the isolation of this compound from another rhododendron, *R. decorum* [20].

A similar metabolite, myricetin, was previously detected in *Rhododendron* species, i.e., *R. adamsii*, *R. sichotense*, *R. ambiguum*, and *R. luteum* [8,14,21]. Its high concentration of 1.377 ± 0.012 mg/g DE) was similar to the amount determined in the leaves of *R. adamsii* [21]. Quercetin was another aglycone present in a substantial amount in RL-M (1.609 ± 0.011 mg/g DE). This observation is in agreement with previous studies, showing that quercetin and its glycosides commonly occur in *Rhododendron* species [1,22]. Moreover, an LC-MS analysis revealed a small amount of 3-*O*-methylquercetin in RL-M. Among flavonoid aglycones, trace amounts of luteolin, apigenin, kaempferol, and laricitrin were also detected. All flavonoid aglycones detected in the *R. luteum* methanolic extract (except for taxifolin) were present in higher concentrations than previously reported [9]. This is probably a result of plant material deballasting in the Soxhlet apparatus and the exhaustive extraction procedure at low temperature (25 °C). Additionally, the sample preparation method applied in this study allowed us to quantify eriodictyol and naringenin.

Our study indicated quercetin derivatives, i.e., hyperoside, isoquercetin, and quercitrin, as the main flavonoid glycosides occurring in RL-M (respectively: 9.044 ± 0.000; 8.980 ± 0.086; 1.822 ± 0.024 mg/g DE). Another detected compound, afzelin, had not been previously observed in *R. luteum* extracts; while present, its concentration was found to be relatively low (0.018 ± 0.002 mg/g DE) in our study. Glycosides found in trace amounts comprised vitexin with isovitexin and naringenin 7-*O*-glucoside.

**Figure 2:**
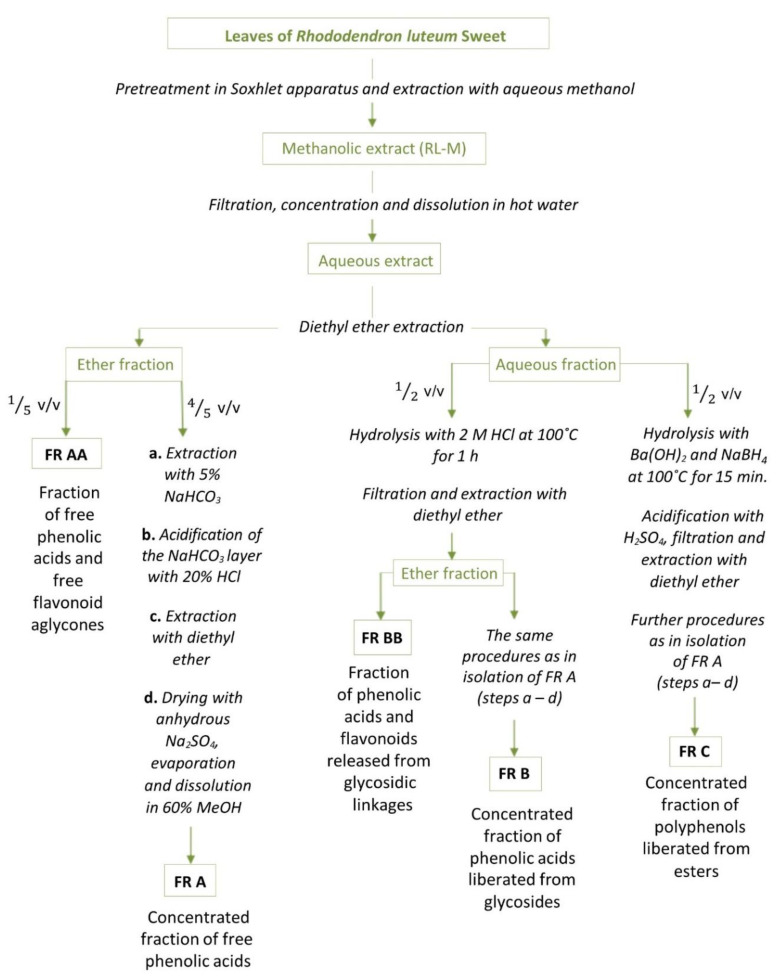
General scheme of the sample preparation procedures.

The optimized Ibrahim and Towers procedure was applied to obtain fractions of phenolic acids and flavonoids (see Figure 2) [12]. As a result, a purified and concentrated fraction of free phenolic acids was obtained (FR A). In this fraction, ferulic acid was present in a large amount, i.e., 40.375 ± 1.055 mg/g DE, whereas its concentration in RL-M was low. Protocatechuic acid was the second most dominant phenolic acid present in FR A (19.141 ± 0.154 mg/g DE). It was also detected in Himalayan rhododendrons, although its amount there was significantly lower [23]. Among free phenolic acids, *p*-coumaric and gallic acids were found in substantial amounts, i.e., 5.891 ± 0.155 and 3.383 ± 0.000 mg/g DE, respectively. The content of gentisic acid (0.667 ± 0.002 mg/g DE) was relatively low, as seen in the other fractions as well. Similarly to RL-M, 4-hydroxybenzoic and caffeic acids were below the quantification limit, whereas vanillic acid was not detected in FR A.

Acidic hydrolysis revealed the presence of high amounts of caffeic acid, which occurs in plant material mostly as glycosides (33.056 ± 0.042 mg/g DE). Shrestha et al. (2017) showed that glycosidically-bound caffeic acid is characteristic of *Rhododendron* species. As reported in their paper, eight different caffeoyl hexosides were determined in the *Rhododendron* genus [18]. Gallic acid was the second most abundant compound in FR B (8.200 ± 0.384 mg/g DE). Other phenolics found in substantial amounts were protocatechuic and gentisic acid. The extraction procedure used in this study yielded higher amounts of these compounds in comparison with their concentration in Himalayan rhododendrons [23]. Additionally, 4-hydroxybenzoic, vanillic, *p*-coumaric, and ferulic acids were identified in FR B, but below their quantification limits. Among flavonoid aglycones, dihydromyricetin, catechin, and eriodictyol were concentrated during the extraction procedure and were found in higher amounts than in FR BB.

The only phenolic acids released after alkaline treatment were *p*-coumaric (146.840 ± 1.485 mg/g DE) and gentisic acids (trace amounts). It is probable that *p*-coumaric acid was released from chlorogenic acids, which are esters of hydroxycinnamic acids with quinic acid occurring in rhododendron species [18]. Moreover, relatively low amounts of dihydromyricetin and quercetin were detected in FR C.

The modifications of the Ibrahim and Towers method proposed in some previous studies (Olech et al. 2020) involved obtaining polyphenolic fractions which contained high concentrations of free flavonoid aglycones with free phenolic acid (FR AA) and flavonoids, as well as the phenolic acids released from their glycosides (FR BB) [13].

As can be seen in Table 1, fraction AA contained a huge amount of catechin (55.678 ± 0.155 mg/g DE). Moreover, the extraction of this fraction revealed a high concentration of free myricetin (15.293 ± 0.802 mg/g DE) in the plant material. Other aglycones present in large amounts in FR AA were dihydromyricetin (91.923 ± 4.678 mg/g DE) and quercetin (23.000 ± 0.979 mg/g DE). Among free flavonoids, relatively small amounts of taxifolin (0.928 ± 0.031 mg/g DE) and eriodictyol (0.480 ± 0.005 mg/g DE) were quantified. Naringenin and luteolin were detected, although their concentrations were below the quantification limit, whereas laricitrin was not observed in FR AA.

As mentioned above, FR BB was collected after acidic hydrolysis to analyze the flavonoid aglycones present in the raw material in the form of glycosides. As shown in Table 1, the prevailing flavonoid aglycone was quercetin, which commonly occurs in rhododendrons in the glycosidic form. Its concentration of 84.270 ± 1.589 mg/g DE was approximately seven times higher than in *R. adamsii* [19]. Moreover, FR BB contained the highest amount of myricetin (56.404 ± 1.589 mg/g DE), which is also higher than the level observed in *R. adamsii* leaf extracts [19]. The contents of other aglycones, i.e., dihydromyricetin, catechin, and eriodictyol, were significantly lower (respectively: 2.637 ± 0.085; 6.869 ± 0.184; 0.188 ± 0.011 mg/g DE).

### 2.2. Antioxidant and Anti-Inflammatory Activity of Polyphenolic Fractions

Polyphenols may exert an anti-inflammatory effect. It has been reported that different phenolic acids and flavonoids have anti-inflammatory activity, e.g., by modulating the enzymes involved in inflammation responses, such as cyclooxygenases (COXs) and lipoxygenases (LOXs) [24]. Therefore, we decided to examine the anti-inflammatory potential of RL and its polyphenolic fractions in in vitro assays (inhibition of COX 1, COX 2, and LOX activity).

All samples were tested in the anti-COX assay, although only RL-M and FR AA exhibited moderate activity. As shown in Table 2, RL-M inhibited both isoforms of cyclooxygenase (IC_50_ values: 311.18 ± 10.95 and 608.09 ± 14.78 µg DE/mL for the COX 1 and COX 2 assays, respectively). Among the many health-beneficial polyphenols present in RL-M, 5-*O*-caffeoylquinic acid was the dominant compound. According to scientific papers on the anti-inflammatory activity of hydroxycinnamic acids, which includes COX inhibition, it can be assumed that the high level of 5-*O*-caffeoylquinic acid in RL-M strongly influenced the observed activity [25,26,27]. Interestingly, COX 2 was also inhibited by FR AA (IC_50_ 53.40 ± 5.07 µg DE/mL), which also contained considerable amounts of 5-*O*-caffeoylquinic acid. Moreover, it comprised large amounts of catechin, dihydromyricetin, and quercetin, which have also been reported to ameliorate inflammation [28,29,30]. This conclusion seems to be in agreement with previous observations showing a synergistic anti-inflammatory effect of catechin and quercetin [31].

As can be seen in Table 3, all samples had high anti-LOX activity in comparison to the positive control, i.e., acetylsalicylic acid. The IC_50_ values in FR B and FR BB ranged from 0.33 ± 0.01 to 2.96 ± 0.06 mg DE/mL, respectively. The strongest LOX inhibitory capacity of FR B may be related to the high content of constituents such as gallic acid, protocatechuic acid, caffeic acid, and quercetin, which have proven anti-LOX activity [24,32,33,34]. On the other hand, the activity of FR C (which was one of strongest LOX inhibitors) is probably related to the high *p*-coumaric acid content. This phenolic acid was previously reported to exhibit anti-LOX activity [35]. In turn, RL-M, FR AA, and FR BB, containing lower amounts of phenolic acids, exhibited lower activity. It is interesting to note that our results are in agreement with data showing a correlation between the total phenolic content and the ability to inhibit lipoxygenase [34]. Similar results to those for RL-M in the anti-LOX assay were obtained in the case of the *R. arboreum* methanolic extract [36].

Another enzyme involved in the inflammation process is hyaluronidase. Due to the degradation of the hyaluronic acid present in the extracellular matrix, hyaluronidase increases the permeability of soft tissues during inflammation. Thus, its inhibitors could be used as potential anti-inflammatory agents [37,38]. Since polyphenol-rich extracts can inhibit hyaluronidase, we decided to test all samples with an antihyaluronidase assay [4]. Epigallocatechin gallate (EGCG), known for its effect on hyaluronidase, was used as a standard [39].

As shown in our findings, the four samples exhibited high hyaluronidase inhibition capacity. The IC_50_ values obtained in the assay ranged from 78.76 ± 2.09 to 429.07 ± 31.08 µg/mL (Table 3). Only FR A and FR BB had low activity and, due to the intense color of the concentrated samples, it was impossible to determine their IC_50_ values with the use of the spectrophotometric method.

Taking into account the relatively low IC_50_ value of the standard, the active samples might be considered as potent hyaluronidase inhibitors. The methanolic extract contained great abundance of polyphenolics, including chlorogenic acid, which has been reported to induce antihyaluronidase effect [40]. In addition to phenolic acids and flavonoids, the extract may have contained other hyaluronidase-inhibiting compounds like tannins, procyanidins, or triterpene saponins, although further research is needed to determine the contents of the RL extract in more detail [4,41]. Moreover, it has previously been suggested that complex synergistic interactions between plant metabolites, rather than a particular group of compounds acting alone, are responsible for the high antihyaluronidase activity [40]. However, as demonstrated in our results, polyphenols significantly contribute to the activity exhibited by RL-M. As can be seen, particular condensed fractions had higher activity than basic methanolic extract. This is in agreement with the correlation between phenolic content and hyaluronidase inhibitory activity reported by Kolayli et al. [4].

Polyphenols have different mechanisms of antioxidant action [42]. Thus, we decided to determine the antiradical capacity of the RL samples in an ABTS assay based on one electron donation, and an ORAC assay based on hydrogen atom transfer [43,44]. Since the presence of polyphenols influences the chelating capacity of extracts, we also decided to determine the chelating power of the RL samples. The results of all tests were expressed as equivalents of standard compounds, i.e., the amount of the standard that has activity equivalent to that of the extract.

The antioxidant activity of *R. luteum* leaf extracts was previously determined via ABTS assay, although the current results have approximately tenfold higher values [8]. The better results obtained in this research in comparison to our previous study may be related to the sample preparation procedure, which included deballasting in the Soxhlet apparatus. The antiradical potential of RL-M may have resulted from the high abundance of different phenolics, as well as the probable presence of other radical scavengers, e.g., tannins or triterpenes. Additionally, RL-M may have contained small amounts of carotenoids, which are effective antioxidant metabolites [45].

We have observed differences in the activity of the RL polyphenolic fractions, which were undoubtedly related to the differences in their composition. The results of the antiradical activity in FR BB and FR C, as determined in the ABTS test, ranged from 0.62 ± 0.03 to 5.09 ± 0.23 g/g DE, respectively (Table 4). The TE values of fractions confirmed the high scavenging ability of their constituents, particularly phenolic acids. The high radical reducing power of *p*-coumaric acid, which is the most abundant in FR C, was previously observed by Kilic et al. [46]. The similar activity of FR B may be related to the presence of caffeic acid, whereas the slightly less active FR A contained another radical scavenger, ferulic acid [47,48]. FR AA and BB, which did not contain those constituents, exhibited lower activity.

As presented in Table 4, there were considerable differences between the samples in the ORAC test. The results varied from 5.76 ± 0.03 to 221.81 ± 20.19 g/g DE for RL-M and FR B, respectively. As indicated above, the most active FR B contained polyphenolic acids which had been released from glycosides. Caffeic acid was the most abundant compound in this group. Its excellent ability to quench free radicals was previously determined in a crocin bleaching assay, which is similar to the ORAC test, and in an ABTS radical scavenging analysis [47]. In the cited study, the second most dominant gallic acid was also indicated as a very active scavenger. Slightly lower antioxidant power was exhibited by FR A, which contained mostly ferulic and protocatechuic acids. Both compounds were classified as effective radical scavengers based on the results of the ORAC test [48,49]. Among flavonoid aglycones known for their antiradical capacity, dihydromyricetin, catechin, and quercetin were present in substantial amounts in the most active FR B and FR A [29,31].

The results of the antioxidant assays are in agreement with studies reporting a strict correlation between polyphenolic compounds detected in extracts and their antioxidant activity [9]. Such a correlation was also observed in previous investigations of *R. luteum* leaves [8]. As suggested by the authors of that report, the activity of phenolic-rich samples may be additionally related to some synergistic action between particular polyphenols.

As seen in Table 4, the ion chelating ability of the polyphenol-rich samples was quite marginal, which is in agreement with our previous study [8,9]. The results were in the range from 4.73 ± 0.33 to 521.81 ± 26.22 mg of Na_2_EDTA/g DE for RL and FR C, respectively.

## 3. Materials and Methods

### 3.1. Chemicals and Apparatus

Analytical standards of taxifolin, luteolin, 3-*O*-methylquercetin, kaempferol, hyperoside, rutin, isorhamnetin, isokaempferide, rhamnetin, sakuranetin, chrysin, prunetin, rhamnazin, morin, isoquercetin, vitexin, isovitexin, quercitrin, kaempferitrin, luteoloside, eriodictyol-7-*O*-glucopyranoside, narirutin, naringin, apigetrin (apigenin 7-glucoside), 5-*O*-caffeoylquinic acid, 4-*O*-caffeoylquinic acid, 3-*O*-caffeoylquinic acid, gallic acid, protocatechuic acid, 4-hydroxybenzoic acid, 3-hydroxybenzoic acid, salicylic acid, rosmarinic acid, caffeic acid, vanillic acid, syringic acid, *p*-coumaric acid, *m*-coumaric acid, *o*-coumaric acid, 3,4-dimethoxycinnamic acid, sinapic acid, isoferulic acid, and ferulic acid as well as Trolox, LC grade methanol, acetonitrile, 2-methylpropionamide dihydrochloride (AAPH), ferrozine (3-(2-pyridyl)-5,6-bis-(4-phenyl-sulfonic acid)-1,2,4-triazine), 2,20-azino-bis-3(ethylbenzthiazoline-6-sulphonic acid) (ABTS), lipoxygenase, di-sodium tetraborate, linoleic acid, sodium phosphate monobasic solution, sodium phosphate dibasic solution, bovine serum albumin, hyaluronic acid, sodium chloride solution, sodium acetate, acetic acid, and hyaluronidase were obtained from Sigma-Aldrich Fine Chemicals (St. Louis, MO, USA). Naringenin, astragalin, apigenin, tiliroside, nicotiflorin, and fluorescein sodium salt were purchased from Roth (Karlsruhe, Germany). Afzelin and dihydromyricetin were supplied by LGC (LGC Group, Teddington, UK). Laricitrin was purchased from Extrasynthese (Lyon, France). Quercetin was obtained from Fluka (Buchs, Switzerland). Catechin, luteolin-7-*O*-glucoside, luteolin 3,7-diglucoside, naringenin 7-*O*-glucoside, eriodictyol, sinapic acid, gentisic acid, and myricetin were supplied by ChromaDex (Irvine, CA, USA). Iron (II) chloride (FeCl_2_) and boric acid were purchased from Avantor Performance Materials Poland S.A. (Gliwice, Poland). The COX (ovine/human) Inhibitor Screening Assay Kit was purchased from Cayman Chemical (Ann Arbor, MI, USA). A Millipore Direct-Q3 purification system (Bedford, MA, USA) was used to prepare LC-MS grade water.

A Heidolph Basis Hei-VAP Value evaporator (Schwabach, Germany) was used for evaporation of all samples. Lyophilization was performed in the Free Zone 1 apparatus (Labconco, Kansas City, KS, USA). Spectrophotometric measurements were conducted using an Infinite Pro 200F microplate reader from Tecan Group Ltd. (Männedorf, Switzerland) with the use of black or transparent 96-well microplates (Nunclon, Nunc; Roskilde, Denmark).

### 3.2. Plant Material

Leaves of *Rhododendron luteum* Sweet were collected in the Kołacznia nature reserve (Nowa Sarzyna, Poland; 50°18′21.6″ N 22°16′01.2″ E) in June 2019 with RDOS (Regional Director for Environmental Protection) permission (No. WPN.6205.45.2019.ŁL.2). The leaves were air-dried at ambient temperature and stored in the Department of Pharmaceutical Botany, Medical University of Lublin, Poland. The voucher specimen number RL-01/19 was deposited.

### 3.3. Sample Preparation

The ground plant material (60 g) was extracted with petroleum for seven days and with chloroform for another seven days in a Soxhlet apparatus. This ensured the removal of waxes, chlorophyll, oils, and other ballast substances. To obtain fractions of free and bound phenolic acids and flavonoid aglycones, procedures based on previous experiments were used [12,13]. A 56-g portion of the pretreated dry plant material was extracted in a shaking water bath three times with 350 mL of 70% and three times with 350 mL of 90% methanol (each time for 60 min) at 25 °C. The collected extracts were concentrated. A precisely measured aliquot (10 mL) of the methanolic extract was taken as an RL-M sample. The rest (190 mL) was evaporated under lowered pressure, dissolved in hot water, cooled, and filtered. Then, exhaustive extraction with diethyl ether was applied. Free phenolic acids and free flavonoid aglycones passed to the ether fraction. The acidic and alkaline hydrolyses preformed in the water layer yielded fractions for analyses of bound phenolic acids and bound aglycones (compounds are liberated from their glycosides or esters during hydrolysis). A scheme of all applied procedures is presented in Figure 2, and extraction efficiencies are given in Table 5.

### 3.4. LC-ESI(−)-MS/MS Analysis

The LC-MS/MS system consisted of an Agilent 1200 Series chromatograph (Agilent Technologies, Santa Clara, CA, USA) and a 3200 QTRAP linear ion trap quadrupole mass spectrometer equipped with a Turbo V™ source and an electrospray ionization (ESI) probe (Sciex, Redwood City, CA, USA). Separations were carried out on an Eclipse XDB-C18 analytical column (4.6 × 150 mm, 5 µm; Agilent Technologies, USA). The previously used LC-MS methods were combined and modified to analyze a broader range of phenolic/polyphenolic analytes [9,50]. The chromatographic conditions were the same as those described by Olech et al. 2020 [50]. The total separation time was 28 min. Detection and qualification were performed on a 3200 QTRAP mass spectrometer with a negative ion multiple reaction monitoring (MRM) mode. The optimized mass analyzer settings, i.e., MRM transitions, declustering potential, entrance potential, collision cell exit potential, collision energy, and the selection of product ions were determined experimentally for each compound (Table S1 in the Supplementary material). The instrument parameters were as follows: ion spray voltage at -4500 V, temperature at 500 °C, CUR gas at 23 psi, nebulizer (gas1) and heater (gas2) at 50 and 60 psi. MS data acquisition and processing were performed using Analyst 1.5 software (AB Sciex, Redwood City, CA, USA). The compounds were quantified on the basis of peak areas of the most intense MRM transitions using the results from calibration curves generated for the corresponding standards. The LOD (limit of detection) and LOQ (limit of quantification) values were established at a signal-to-noise ratio of 5:1 and 10:1, respectively (Table S2; Supplementary material). The LC-MS assay was performed at least three times for each standard solution and sample. The samples were filtered through a hydrophilic polytetrafluoroethylene (PTFE) 0.20 μm membrane (Merck, Darmstadt, Germany) syringe filter prior to LC injection.

### 3.5. Inhibition of Lipoxygenase (LOX) Activity

The lipoxygenase inhibition activity was assayed according to a method described by Nowacka-Jechalke et al., with slight modifications [51]. Briefly, a mixture of 100 µL of boric acid buffer (0.2 M, pH 9), 10 µL of LOX (2660 U/mL), and 10 µL of the tested sample was incubated for 2 min at 37 °C. After this time, 40 µL of linoleic acid (0.15 mg/mL) was added. Absorbance was measured at 234 nm immediately after substrate addition and again after 3 min of incubation. During the reaction, conjugated *cis*, *trans*-hydroperoxydiene is formed from linoleic acid. Acetylsalicylic acid (ASA) was used as a positive control. The corresponding control contained the same concentration of LOX in the absence of the inhibitor. The percentage of inhibition was determined using the following equation:

% inhibition = (ΔABLK − ΔAP)/ΔABLK × 100%,

where ΔABLK is the absorbance increment of the blank sample and ΔAP is the increment of the tested sample. At least six dilutions of each extract were examined to plot a dose-response curve and determine the half maximal inhibitory concentration (IC_50_). All measurements were made in triplicate and averaged.

### 3.6. Inhibition of Cyclooxygenase (COX) Activity

The anti-inflammatory activity of the samples was examined using a COX (ovine/human) Inhibitor Screening Assay Kit (Cayman Chemical). All samples were examined according to the instruction provided by the producer. Primary solutions of examined extracts were dissolved in 80% methanol. Measurements were performed in triplicate for each sample and averaged. At least five dilutions of each extract and the positive control were examined to plot a dose-response curve and determine the half maximal inhibitory concentration (IC_50_). All measurements were made in triplicate and averaged.

### 3.7. Inhibition of Hyaluronidase Activity

The antihyaluronidase activity was evaluated with the method proposed by Yahaya et al., adapted to the microscale [52]. All extracts and the positive control were diluted in 80% methanol. Aliquots of 20 µL of the examined samples were mixed with 20 µL of phosphate buffer (20 mM; with 77 mM NaCl and 0.1 mg/mL of albumin; pH 7; 37 °C) and 20 µL of the hyaluronidase enzyme (40–100 U/mL in the same phosphate buffer). The mixtures were incubated at 37 °C for 10 min. Then, 20 µL of hyaluronic acid (0.5 mg/mL in 300 mM sodium phosphate buffer; pH 5.35; 37 °C) was added. After 45 min of incubation at 37 °C, undigested hyaluronic acid was precipitated with 100 µL of acid albumin solution (2 mg/mL in 79 mM acetic acid with 24 mM sodium acetate; pH 3.75; 25 °C). The mixture was shaken for 10 min at 25 °C. After this time, the transmittance was measured at a wavelength of 600 nm. Epigallocatechin gallate (EGCG) was the standard substance used in this experiment as a positive control. The percentage of hyaluronidase inhibition was calculated using the following formula:

% inhibition = (TS − TBLK)/(TC − TBLK) × 100%,

where TS is the transmittance of the examined sample, TBLK is the transmittance of the negative control (containing buffer instead of the sample), and TC is the transmittance of the mixture containing buffer instead of the enzyme. At least six dilutions of each extract were examined to plot a dose-response curve and determine the half maximal inhibitory concentration (IC_50_). All measurements were made in triplicate and averaged.

### 3.8. ORAC

The oxygen radical absorbance capacity assay was performed according to the method described by Olech et al. [9]. All extracts and reagents were diluted in freshly prepared phosphate buffer (75 mM, pH 7.4). Briefly, 10 µL of the sample was mixed with 150 µL of fluorescein (10 nM) and incubated for 20 min at 37 °C. In order to initiate the reaction, 25 µL of AAPH (240 mM) was added. The fluorescence was measured after every 90 s for 120 min (excitation wavelength – 485 nm; emission wavelength – 515 nm). All determinations were conducted in triplicate and averaged. The activity of each extract was expressed as g of Trolox/g of dry extract (DE).

### 3.9. ABTS

The antiradical activity was determined using ABTS (2,2′-azino-bis(3-ethylbenzothiazoline-6-sulfonic acid)) radical according to the method described by Olech et al. [53]. The ABTS stock solution was made by mixing 4 mg of ABTS (aqueous; 9.3 mM) and 1 mg of potassium persulfate (36.6 mM). The mixture was left for 16 h in the dark. The working solution of ABTS was freshly prepared by diluting 0.5 mL of the stock solution with 19.5 mL of methanol. The reacting mixture contained 20 µL of the examined sample and 180 µL of the ABTS working solution. The blank sample contained methanol instead of the extract. Absorbance was measured after 6 min of incubation at 734 nm. Triplicate measurements were taken for each sample and averaged. The percentage of reduction was calculated according to the following formula:

% reduction = (AC − AE)/AC × 100%,

where AC is the absorbance of the control and AE is the absorbance of the extract.

The results were expressed as Trolox equivalents (g of Trolox/g DE) using a standard curve prepared in the same conditions for a series of dilutions of the Trolox solution.

### 3.10. Metal Chelating Activity

The metal chelating activity was examined according to the same modified Guo et al. method used in the previous study [9,54]. Aliquots of 100 µL of the samples were mixed with 25 µL of FeCl_2_ (0.4 mM). After 2 min of pre-incubation at room temperature, 50 µL of ferrozine (1 mM) was added. The mixture was shaken for 1 min and left standing for 9 min. After this time, absorbance was measured at 562 nm. Measurements for each sample were made in triplicate and averaged.

The metal chelating power was determined using the following equation:

% chelating = (1− As/Ab) × 100

where As is the absorbance of the sample and Ab is the absorbance of the blank. The results were expressed as mg of Na_2_EDTA/g DE.

### 3.11. Statistical Analysis

All tests were performed in triplicate, and the results are expressed as a mean with standard deviation (SD). Statistical analyses were carried out using STATISTICA 10.0 (StatSoft Poland, Cracow, Poland).

## 4. Conclusions

The present research is the first report on the detailed phenolic profile of *R. luteum* leaves, including free and bound phenolic acids and flavonoids. As revealed by the LC-ESI-MS/MS-MRM analysis, the overwhelming majority of the studied metabolites in RL occur in free or glycosidically bound forms, whereas protocatechuic acid, ferulic acid, dihydromyricetin, catechin, and taxifolin are largely unbound. The analysis of the fractions after alkaline hydrolysis indicated the presence of esterically-bonded *p*-coumaric acid, gentisic acid, dihydromyricetin, and quercetin. RL was found to contain large quantities of myricetin, dihydromyricetin, quercetin, 3-*O*-methylquercetin, and catechin glycosides. It is worth noting that our study is the first to report the presence of dihydromyricetin, afzelin, and laricitrin in RL leaves. It was indicated that the hydrolytic treatment released a high portion of biologically-active polyphenols from this plant material. This phytochemical data may be of great importance for the effective use of RL as a source of phenolic compounds for potential commercial use.

Our study has also expanded the knowledge of the anti-inflammatory potential of RL, proving the impact of its polyphenols on the activity of enzymes (COX 1, COX 2, hyaluronidase) involved in the inflammatory process. The current study confirmed the assumption of the high anti-LOX and antioxidant activity of the polyphenols present in RL samples. Moreover, particular constituents (5-*O*-caffeoylquinic, caffeic, *p*-coumaric, gallic, protocatechuic acid quercetin, catechin, and dihydromyricetin), which may have an influence on the ability to inhibit enzymes and act as a radical scavengers, were indicated.

## Figures and Tables

**Table 1:** Content of free and bound phenolic acids, flavonoid aglycones, and glycosides (mg/g of dry extract). Mean values of three replicated assays and standard deviations. Abbreviations: RL-M—methanolic extract, FR A—fraction of free phenolic acids, FR B—fraction of glycoside-bound phenolic acids, FR C—fraction of ester-linked phenolic acids, FR AA—fraction containing free phenolic acids and free flavonoid aglycones, FR BB—fraction containing glycoside-bound phenolic acids and flavonoid aglycones, BQL—compound detected, but its concentration was below the quantification limit.

	RL-M	FR A	FR B	FR C	FR AA	FR BB
Phenolic acids						
gallic	0.267 ± 0.001	3.383 ± 0.000	8.200 ± 0.384	0	3.376 ± 0.059	0.777 ± 0.003
protocatechuic	0.397 ± 0.002	19.141 ± 0.154	5.160 ± 0.170	0	4.250 ± 0.026	4.730 ± 0.048
4-hydroxybenzoic	BQL	BQL	BQL	0	BQL	BQL
gentisic	0.016 ± 0.001	0.667 ± 0.002	2.248 ± 0.056	BQL	0.335 ± 0.003	0.162 ± 0.007
caffeic	BQL	BQL	33.056 ± 0.042	0	0	0
vanillic	0	0	BQL	0	0	BQL
*p*-coumaric	0.087 ± 0.003	5.891 ± 0.155	BQL	146.840 ± 1.485	5.293 ± 0.181	1.551 ± 0.074
ferulic	BQL	40.375 ± 1.055	BQL	0	BQL	BQL
5-*O*-caffeoylquinic	18.546 ± 0.093	0	0	0	11.191 ± 0.526	1.258 ± 0.057
Flavonoid aglycones						
laricitrin	BQL	0	0	0	0	BQL
dihydromyricetin	4.032 ± 0.086	102.912 ± 4.720	29.120 ± 2.712	1.595 ± 0.007	91.923 ± 4.678	2.637 ± 0.085
catechin	6.081 ± 0.043	51.278 ± 0.402	26.720 ± 0.904	0	55.678 ± 0.155	6.869 ± 0.184
naringenin	0.588 ± 0.005	0	0	0	BQL	BQL
taxifolin	0.019 ± 0.000	2.675 ± 0.044	BQL	0	0.928 ± 0.031	BQL
myricetin	1.377 ± 0.012	BQL	BQL	0	15.293 ± 0.802	56.404 ± 1.589
luteolin	BQL	BQL	BQL	0	BQL	BQL
eriodictyol	0.113 ± 0.001	0.844 ± 0.014	0.637 ± 0.030	0	0.480 ± 0.005	0.188 ± 0.011
quercetin	1.609 ± 0.011	23.722 ± 0.603	34.160 ± 0.340	29.210 ± 0.370	23.000 ± 0.979	84.270 ± 1.589
3-*O*-methylquercetin	0.013 ± 0.000	0	0	0	0.304 ± 0.015	0.574 ± 0.001
apigenin	BQL	BQL	BQL	0	BQL	BQL
kaempferol	BQL	BQL	BQL	BQL	BQL	BQL
Flavonoid glycosides						
afzelin	0.018 ± 0.002	0	0	0	0	0
hyperoside	9.044 ± 0.000	0	0	0	0	0
isoquercetin	8.980 ± 0.086	0	0	0	0	0
vitexin	BQL	0	0	0	0	0
isovitexin	BQL	0	0	0	0	0
quercitrin	1.822 ± 0.024	0	0	0	0	0
naringenin 7-*O*-glucoside	BQL	0	0	0	0	0

**Table 2:** Anti-inflammatory activity of *Rhododendron luteum* Sweet leaf samples. The inhibitory effects on cyclooxygenase 1 (COX 1) and cyclooxygenase 2 (COX 2) were expressed as IC_50_ (concentration of the sample (µg DE/mL) which produces 50% of COX inhibition). Abbreviations: ASA – acetylsalicylic acid, other abbreviations as in Table 1.

Sample	COX 1 Inhibitory Activity IC_50_ (µg/mL)	COX 2 Inhibitory Activity IC_50_ (µg/mL)
RL-M	311.18 ± 10.95	608.09 ± 14.78
FR A	0	0
FR B	0	0
FR C	0	0
FR AA	0	53.40 ± 5.07
FR BB	0	0
ASA	14.80 ± 0.27	35.0 ± 2.93

**Table 3:** Antihyaluronidase and antilipoxygenase activity of polyphenolic fractions from *Rhododendron* luteum Sweet leaves. The inhibition of enzymes is expressed as the IC_50_ value (concentration of the sample which causes 50% inhibition). Abbreviations: n. a.—not analyzed, EGCG—epigallocatechin gallate, ASA—acetylsalicylic acid, other abbreviations as in Table 1.

Sample	Lipoxygenase IC_50_ (mg DE/mL)	Hyaluronidase IC_50_ (µg DE/mL)
**RL-M**	2.27 ± 0.01	305.21 ± 3.21
**FR A**	1.11 ± 0.07	>1000
**FR B**	0.33 ± 0.01	97.44 ± 4.98
**FR C**	0.40 ± 0.01	78.76 ± 2.09
**FR AA**	2.54 ± 0.14	429.07 ± 31.08
**FR BB**	2.96 ± 0.06	>1000
**EGCG**	n. a.	276.50 ± 1.34
**ASA**	2.12 ± 0.13	n. a.

**Table 4:** Antioxidant activity and metal chelating power of *Rhododendron luteum* Sweet leaf samples. The results of the radical scavenging assay with ABTS and ORAC are expressed as g of Trolox/g of dry extract (DE). The results of metal chelating activity are expressed as mg of Na_2_EDTA/g DE. Abbreviations as in Table 1.

Sample	ABTS (g Trolox/g)	ORAC (g Trolox/g)	Chelating Power (mg Na_2_EDTA/g)
**RL-M**	4.67 ± 0.00	5.76 ± 0.03	4.73 ± 0.33
**FR A**	3.03 ± 0.04	175.32 ± 9.35	128.54 ± 10.444
**FR B**	4.18 ± 0.04	221.81 ± 20.19	292.36 ± 39.72
**FR C**	5.09 ± 0.23	91.19 ± 0.50	521.81 ± 26.22
**FR AA**	1.68 ± 0.01	107.14 ± 1.48	80.39 ± 6.35
**FR BB**	0.62 ± 0.03	40.87 ± 0.14	42.78 ± 0.44

**Table 5:** Extraction efficiencies for RL polyphenolic samples (% w/w of dry plant weight). Abbreviations as in Table 1.

Sample	RL-M	FR A	FR B	FR C	FR AA	FR BB
**Extraction Efficiency (%)**	31.18	0.64	0.25	0.20	1.35	2.67

## Data Availability

Not applicable.

## Supplementary Materials

The following supporting information can be downloaded online. Figure S1: LC-MS chromatograms obtained in multiple reaction monitoring (MRM) mode of flavonoid aglycones detected in *R. luteum* leaves (sample RL-M); Figure S2: LC-MS chromatograms obtained in multiple reaction monitoring (MRM) mode of chosen flavonoid glycosides detected in *R. luteum* leaves (sample RL-M); Table S1: Summary of optimized QTRAP parameters for the LC-MS analysis of phenolic acids and flavonoid compounds; Table S2: Analytical parameters used for quantitative determination of phenolic acids and flavonoids detected in samples.

## Sample Availability

Samples are not available from the authors.
