# Supplementary material for: LC-ESI-MS/MS Characterization of Concentrated Polyphenolic Fractions from Rhododendron luteum and Their Anti-Inflammatory and Antioxidant Activities
Source: Molecules. 2022 Jan 26;27(3):827. doi: 10.3390/molecules27030827 (PMC8840727; doi:10.3390/molecules27030827)
Supplement: Supplementary file 1 [file molecules-27-00827-s001.zip › molecules-1558298-supplementary.pdf]

Supplementary material

LC-ESI-MS/MS characterization of concentrated polyphenolic fractions from *Rhododendron luteum* and their anti-inflammatory and antioxidant activities

Lena Łyko , Marta Olech * and Renata Nowak

Department of Pharmaceutical Botany, Medical University of Lublin, 1 Chodźki Street, 20-093 Lublin, Poland; lenalyko18@gmail.com (L.Ł.); renata.nowak@umlub.pl (R.N.)
* Correspondence: marta.olech@umlub.pl (M.O.); Tel.: +48-814-487-060

Table S1. Summary of optimized QTRAP parameters for the LC-MS analysis of phenolic acids and flavonoid compounds. Abbreviations: Q1/Q3 – m/z values for precursor and fragment ion detected in Q1 and Q3 quadrupole, respectively (tracked MRM transitions); declustering potential (DP); entrance potential (EP); collision cell exit potential (CXP); collision energy (CE); collision cell exit potential (CXP).

Compound	Retention time [min]	Q1/Q3 [m/z]	DP [V]	EP [V]	CEP [V]	CE [eV]	CXP [V]
**Phenolic acids**							
Gallic acid	5.16	168.7/78.9	-35	-3	-12	-36	0
		168.7/124.9	-35	-3	-12	-14	0
3-Caffeoylquinic acid	6.93	352.9/191.1	-25	-10	-24.7	-28	-3
		352.9/178.9	-25	-10	-24.7	-22	-3
Protocatechuic acid	8.42	152.9/80.9	-55	-1	-10	-26	0
		152.9/107.8	-55	-1	-10	-38	0
5-Caffeoylquinic acid	9.24	353.0/190.9	-35	-4.5	-16	-20	-2
		353.0/85	-35	-4.5	-16	-60	0
4-Caffeoylquinic acid	9.38	352.9/173	-25	-10	-24.7	-21	-3
		352.9/135	-25	-10	-24.7	-36	-3
		352.9/179			-24.7	-22	-3
4-Hydroxybenzoic acid	10.84	136.8/92.9	-30	-7	-10	-18	0
Gentisic acid	11.37	152.8/80	-70	-4	-16	-110	0
		152.8/96.9	-70	-4	-16	-52	0
Caffeic acid	11.38	178.7/88.9	-30	-6.5	-12	-46	0
		178.7/134.9	-30	-6.5	-12	-16	0
Vanilic acid	11.41	166.8/107.9	-35	-4	-12	-18	0
		166.8/123	-35	-4	-12	-12	0
Syringic acid	11.42	196.9/122.8	-30	-9	-12	-24	0
		196.9/181.9	-30	-9	-12	-12	-2
3-Hydroxybenzoic acid	12.12	136.8/93	-35	-4	-16.7	-16	-2
		136.8/75	-35	-4	-16.7	-48	0
4-Hydroxycinnamic acid (*p*-coumaric acid)	14.10	162.7/119	-30	-8	-12	-14	0
		162.7/93	-30	-8	-12	-44	0

Sinapic acid	14.47	222.8/121	-35	-8.5	-10	-36	0
	14.94	222.8/148.9	-35	-8.5	-10	-20	0
Ferulic acid	14.80	192.8/133.9	-25	-11.5	-14	-16	0
	15.22	192.8/177.9	-25	-11.5	-14	-12	-2
3-Hydroxycinnamic acid (*m*-coumaric acid)	15.50	162.7/119	-35	-4.5	-12	-14	0
		162.7/91	-35	-4.5	-12	-36	0
Rosmarinic acid	15.91	358.7/160.8	-50	-5	-26	-20	-2
		358.7/196.8	-50	-5	-26	-22	-2
2-Hydroxycinnamic acid (*o*-coumaric acid)	16.80	162.7/119	-25	-5	-10	-14	0
		162.7/93	-25	-5	-10	-46	0
3,4-Dimethoxycinnamic acid	17.60	206.9/103	-30	-10	-16	-16	0
		206.9/163	-30	-10	-16	-12	-2
Salicylic acid	17.91	136.8/93	-35	-4	-10	-16	-2
		136.8/75	-35	-4	-10	-48	0
**Flavonoid aglycones**							
Catechin	9.64	288.8/244.9	-45	-4.5	-16	-16	-4
		288.8/109	-45	-4.5	-16	-32	0
Epigallocatechin gallate (EGCG)	11.20	457/169.1	-25	-10	-28.6	-30	-3
		457/125	-25	-10	-28.6	-30	-3
Dihydromyricetin (Ampelopsin; Ampeloptin)	12.10	319/193	-25	-10	-23.5	-30	-3
		319/125	-25	10	-23.5	-30	-3
Naringenin	14.52	270.8/119	-50	-11.5	-12	-34	0
		270.8/150.9	-50	-11.5	-12	-22	0
Taxifolin	15.15	302.7/124.9	-45	-3.5	-18	-26	0
		302.7/284.8	-45	-3.5	-18	-14	-4
Myricetin	16.57	316.7/136.9	-55	-9	-14	-32	0
		316.7/150.9	-55	-9	-14	-26	0
Morin	17.48	300.7/124.9	-50	-3.5	-20	-24	0
		300.7/106.9	-50	-3.5	-20	-30	0
Luteolin	17.82	284.7/132.9	-75	-9	-18	-38	0
		284.7/150.9	-75	-9	-18	-26	0
Eriodictiol	17.89	286.7/134.9	-45	-6	-12	-32	0
		286.7/150.9	-45	-6	-12	-18	-2
Laricitrin (3'-O-Methylmyricetin)	17.9	330.97/151	-25	-10	-23.9	-30	-3
		330.97/315.9	-25	10	-23.9	-30	10
Quercetin	17.94	300.7/150.9	-60	-2.5	-12	-26	0
		300.7/178.8	-60	-2.5	-12	-20	-2
3-*O*-Methylquercetin	18.11	314.7/299.8	-55	-9.5	-22	-18	-4
		314.7/270.8	-55	-9.5	-22	-26	-4
Apigenin	18.64	268.8/117	-70	-9.5	-12	-44	0
		268.8/106.8	-70	-9.5	-12	-34	0
Kaempferol	18.85	284.7/116.8	-70	-5	-12	-46	0
		284.7/93	-70	-5	-12	-52	0
Isorhamnetin	18.99	314.7/299.7	-65	-2.5	-26	-20	-4
		314.7/150.9	-65	-2.5	-26	-30	0

Isokaempferide	19.16	298.8/283.9<br>298.8/226.9	-50<br>-50	-4.5<br>-4.5	-12<br>-12	-18<br>-28	-4<br>-2
Rhamnetin	20.10	314.7/165<br>314.7/120.9	-60<br>-60	-5.5<br>-5.5	-18<br>-18	-24<br>-36	0<br>0
Sakuranetin	21.67	284.7/118.9<br>284.7/164.8	-60<br>-60	-5.5<br>-5.5	-12<br>-12	-34<br>-20	0<br>-2
Chrysin	21.82	252.8/208.9<br>252.8/142.9	-80<br>-80	-10<br>-10	-14<br>-14	-22<br>-26	-2<br>0
Prunetin	21.98	282.8/267.7<br>282.8/238.7	-55<br>-55	-12<br>-12	-18<br>-18	-20<br>-26	-4<br>-2
Rhamnazin	22.37	328.7/270.8<br>328.7/313.8	-70<br>-70	-3<br>-3	-28<br>-28	-26<br>-14	-2<br>-4
			**Flavonoid glycosides**				
Kaempferol 3-rutinoside 4'-glucoside	10.40	754.8/592.8<br>754.8/284.6	-105<br>-105	-3.5<br>-3.5	-20<br>-20	-60<br>-30	-10<br>-22
Kaempferol-3-*O*-galactoside-rhamnoside-7-*O*-rhamnoside (Robinin)	10.80<br>11.07	738.8/592.8<br>738.8/254.8	-90<br>-90	-4.5<br>-4.5	-24<br>-24	-36<br>-80	-24<br>-4
Kaempferol 3-glucoside-7-rhamnoside	11.25	592.7/284.9<br>592.7/430.3	-145<br>-145	-4.5<br>-4.5	-30<br>-30	-42<br>-36	-4<br>-16
Luteolin 3',7'-diglucoside	11.28	609.1/285<br>609.1/447	-70<br>-70	-7.5<br>-7.5	-28<br>-28	-50<br>-32	-4<br>-18
Quercetin 3,7-dirhamnoside	11.35	592.8/445.7<br>592.8/298.9	-90<br>-90	-4<br>-4	-26<br>-26	-48<br>-34	-4<br>-18
Eriodictyol-7-*O*-rutinoside (Eriocitrin)	11.93	594.8/286.9<br>594.8/150.9	-75<br>-75	-4.5<br>-4.5	-28<br>-28	-34<br>-46	-4<br>-2
Quercetin-3-*O*-rutinoside (Rutin)	11.99	608.7/299.6<br>608.7/270.9	-90<br>-90	-8<br>-8	-30<br>-30	-46<br>-60	-4<br>-4
Kempferol 3,7-dirhamnoside (Kaempferitrin)	12.16	576.8/284.8<br>576.8/430.9	-80<br>-80	-4.5<br>-4.5	-28<br>-28	-42<br>-30	-4<br>-18
Apigenin – 6-*C*-glucoside (Isovitexin)	12.38	430.8/310.9<br>430.8/340.9	-65<br>-65	-4.5<br>-4.5	-18<br>-18	-28<br>-26	-4<br>-14
Apigenin – 8-*C*-glucoside (Vitexin)	12.40	430.8/310.9<br>430.8/340.9	-75<br>-75	-4.5<br>-4.5	-20<br>-20	-26<br>-34	-4<br>-14
Quercetin-3-*O*-galactoside (Hyperoside)	12.80	462.7/299.7<br>462.7/254.7	-70<br>-70	-4<br>-4	-18<br>-18	-28<br>-42	-4<br>-2
Luteolin-7-*O*-glucoside (Luteoloside)	12.87	446.8/284.8<br>446.8/132.9	-70<br>-70	-10.5<br>-10.5	-20<br>-20	-30<br>-78	-4<br>0
Quercetin-3-*O*-glucoside (Isoquercetin)	13.00	462.7/299.7<br>462.7/270.7	-85<br>-85	-1.5<br>-1.5	-20<br>-20	-30<br>-44	-4<br>-4
Eriodictyol-7-*O*-glucopyranoside	13.06	448.8/286.9<br>448.8/134.9	-75<br>-75	-4.5<br>-4.5	-20<br>-20	-24<br>-48	-4<br>-2
Kaempferol – 3-*O*-rutinoside (Nicotiflorin)	13.31	592.7/284.8<br>592.7/226.7	-65<br>-65	-12<br>-12	-30<br>-30	-38<br>-68	-2<br>-2
Naringenin-7-*O*-rutinoside (Narirutin)	13.80	578.9/270.8<br>578.9/118.9	-90<br>-90	-4.5<br>-4.5	-24<br>-24	-34<br>-76	-4<br>0

Naringenin-7-*O*-rhamnosidoglucoside (Naringin)	14.50	579.1/151	-80	-4	-26	-54	-2
		579.1/271	-80	-4	-26	-42	-4
Kaempferol – 3-*O*-glucoside (Astragalin)	14.66	446.7/226.8	-75	-9	-20	-54	-2
		446.7/254.8	-75	-9	-20	-40	-2
Quercetin 3-*O*-rhamnoside (Quercitrin)	14.83	446.7/299.7	-65	-9	-18	-30	-4
		446.7/270.7	-65	-9	-18	-40	-4
Apigenin 7-*O*-glucoside (Apigetrin, Cosmosiin)	14.91	430.7/267.7	-70	-9	-20	-38	-4
		430.7/116.9	-70	-9	-20	-84	0
Naringenin 7-*O*-glucoside	15.12	432.7/270.8	-40	-8.5	-20	-22	-4
		432.7/118.9	-40	-8.5	-20	-64	0
Afzelin (Kaempferol 3-rhamnoside)	15.9	431.1/284.9	-25	-10	-27.6	-30	-3
		431.1/254.9	-25	10	-27.6	-30	10
Tiliroside	17.39	592.8/284.8	-70	-7.5	-24	-38	-4
		592.8/254.7	-70	-7.5	-24	-56	-2

Table S2. Analytical parameters used for quantitative determination of phenolic acids and flavonoids detected in samples.

Compound	LOD [ng/mL]	LOQ [ng/mL]	R^2	Linearity range [ng/mL]
**Phenolic acids**				
Gallic acid	1000	1850	0.9986	1850-18500
Protocatechuic acid	200	400	0.9988	1890-18900
5-Caffeoylquinic acid	75	180	0.9991	180-18000
4-Hydroxybenzoic acid	200	250	0.9994	770-19250
Gentisic acid	82	205	0.9992	2050-19900
Caffeic acid	195	389	0.9991	389-19500
Vanilic acid	1000	1830	0.9983	1830-18300
4-Hydroxycinnamic acid (*p*-coumaric acid)	83	200	0.9990	415-13800
Ferulic acid	1250	1830	0.9985	1830-36500
Salicylic acid	500	732	0.9974	1830-18300
**Flavonoid aglycones**				
Catechin	250	330	0.9982	330-6600
Dihydromyricetin (Ampelopsin; Ampeloptin)	45	80	0.9938	30-3000
Naringenin	165	330	0.9980	660-6600
Taxifolin	10	20	0.9981	40-10000
Myricetin	3300	6600	0.9995	6600-66000
Luteolin	6	16	0.9974	33-1650
Eriodictyol	33	66	0.9984	66-6600
Laricitrin (3'-O-Methylmyricetin)	10	20	0.9944	300-10000
Quercetin	66	132	0.9977	132-6600
3-*O*-Methylquercetin	10	12	0.9993	300-30000
Apigenin	15	22	0.9979	89-4470
Kaempferol	33	66	0.9977	165-3300
**Flavonoid glycosides**				
Apigenin – 6-*C*-glucoside (Isovitexin)	100	250	0.9994	1670-50000
Apigenin – 8-*C*-glucoside (Vitexin)	100	200	0.9984	2000-50000

Quercetin-3-*O*-galactoside (Hyperoside)	167	250	0.9983	500-25000
Quercetin-3-*O*-glucoside (Isoquercetin)	167	250	0.9988	2500-50000
Quercetin 3-*O*-rhamnoside (Quercitrin)	50	100	0.9986	1000-25000
Naringenin 7-*O*-glucoside	100	167	0.9987	250-25000
Afzelin (Kaempferol 3-rhamnoside)	10	20	0.9973	30-6000

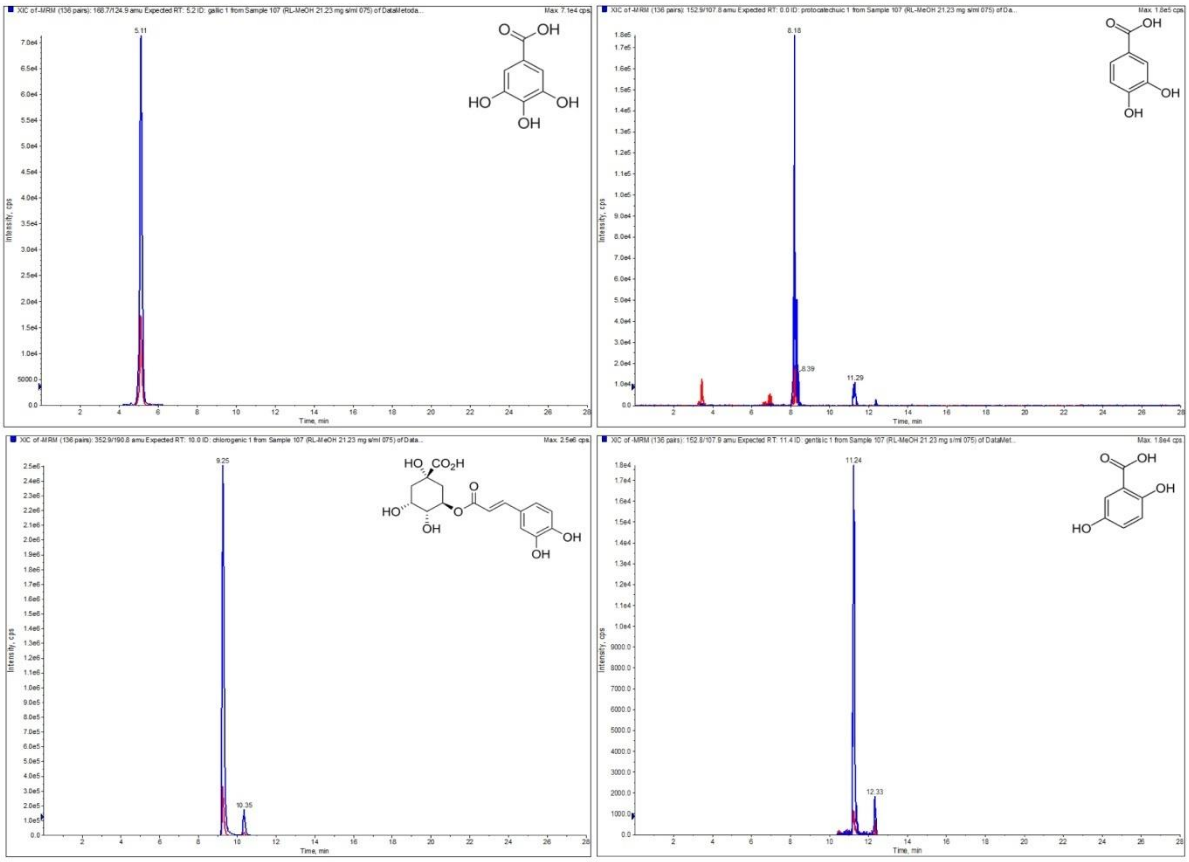

Figure S1. LC-MS chromatograms obtained in multiple reaction monitoring (MRM) mode of flavonoid aglycones detected in R. *luteum* leaves (sample RL-M). Monitored MRM transitions are given in the bracket: gallic acid (*m/z* 168.7→78.9; 168.7→124.9) , protocatechuic acid (*m/z* 152.9→80.9; 152.9→107.8) , 5-O-caffeoylquinic acid (*m/z* 353.0→190.9; 353.0→85) , gentisic acid (*m/z* 152.8→80; 152.8→96.9).

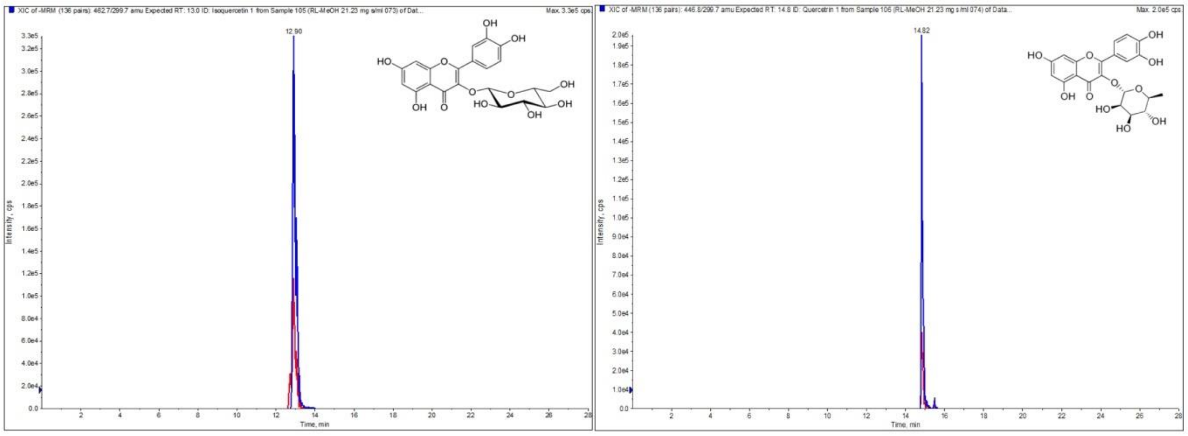

Figure S2. LC-MS chromatograms obtained in multiple reaction monitoring (MRM) mode of chosen flavonoid glycosides detected in R. *luteum* leaves (sample RL-M). Monitored MRM transitions are given in the bracket: isoquercetin (462.7→299.7; 462.7→270.7), quercitrin (446.7→299.7; 446.7→270.7).
